# Improved production of the non-native cofactor F_420_ in *Escherichia coli*

**DOI:** 10.1038/s41598-021-01224-3

**Published:** 2021-11-05

**Authors:** Mihir V. Shah, Hadi Nazem-Bokaee, James Antoney, Suk Woo Kang, Colin J. Jackson, Colin Scott

**Affiliations:** 1 Biocatalysis and Synthetic Biology Team, CSIRO Land and Water, Black Mountain Science and Innovation Precinct, Canberra, ACT Australia; 2 Synthetic Biology Future Science Platform, Black Mountain Science and Innovation Precinct, Canberra, ACT Australia; 3 Research School of Chemistry, Australian National University, Canberra, ACT Australia

**Keywords:** Metabolic engineering, Synthetic biology

## Abstract

The deazaflavin cofactor F_420_ is a low-potential, two-electron redox cofactor produced by some Archaea and Eubacteria that is involved in methanogenesis and methanotrophy, antibiotic biosynthesis, and xenobiotic metabolism. However, it is not produced by bacterial strains commonly used for industrial biocatalysis or recombinant protein production, such as *Escherichia coli*, limiting our ability to exploit it as an enzymatic cofactor and produce it in high yield. Here we have utilized a genome-scale metabolic model of *E. coli* and constraint-based metabolic modelling of cofactor F_420_ biosynthesis to optimize F_420_ production in *E. coli.* This analysis identified phospho-enol pyruvate (PEP) as a limiting precursor for F_420_ biosynthesis, explaining carbon source-dependent differences in productivity. PEP availability was improved by using gluconeogenic carbon sources and overexpression of PEP synthase. By improving PEP availability, we were able to achieve a ~ 40-fold increase in the space–time yield of F_420_ compared with the widely used recombinant *Mycobacterium smegmatis* expression system. This study establishes *E. coli* as an industrial F_420_-production system and will allow the recombinant in vivo use of F_420_-dependent enzymes for biocatalysis and protein engineering applications.

## Introduction

Cofactor F_420_ is required for methanogenesis in Archaea^1–5^, anaerobic oxidation of methane by anaerobic methanotrophs^6–9^, and is involved in secondary metabolism in some Eubacteria^10,11^. As a deazaflavin, it is structurally similar to flavin, but given its lower redox mid-point potential (− 360 mV for F_420_ cf. ~  − 230 mV for flavins) and obligate 2-electron transfer it functions analogously to NAD/NADP^12,13^. It has been suggested that cofactor F_420_-dependent enzymes have significant potential as biocatalysts for the reduction of enoates, imines and ketones, and potentially for other unexplored reactions and processes^14–17^. However, the lack of a cost-effective production system for cofactor F_420_ is a major deterrent in exploring F_420_-dependent reactions for biocatalytic applications. Development of a low-cost production system for F_420_ will be essential for advancing the application of F_420_-dependent enzymes. An ultimate goal of this study is to devise the development of a system for F_420_ production as an end-product and at scale.

**Figure 1:**
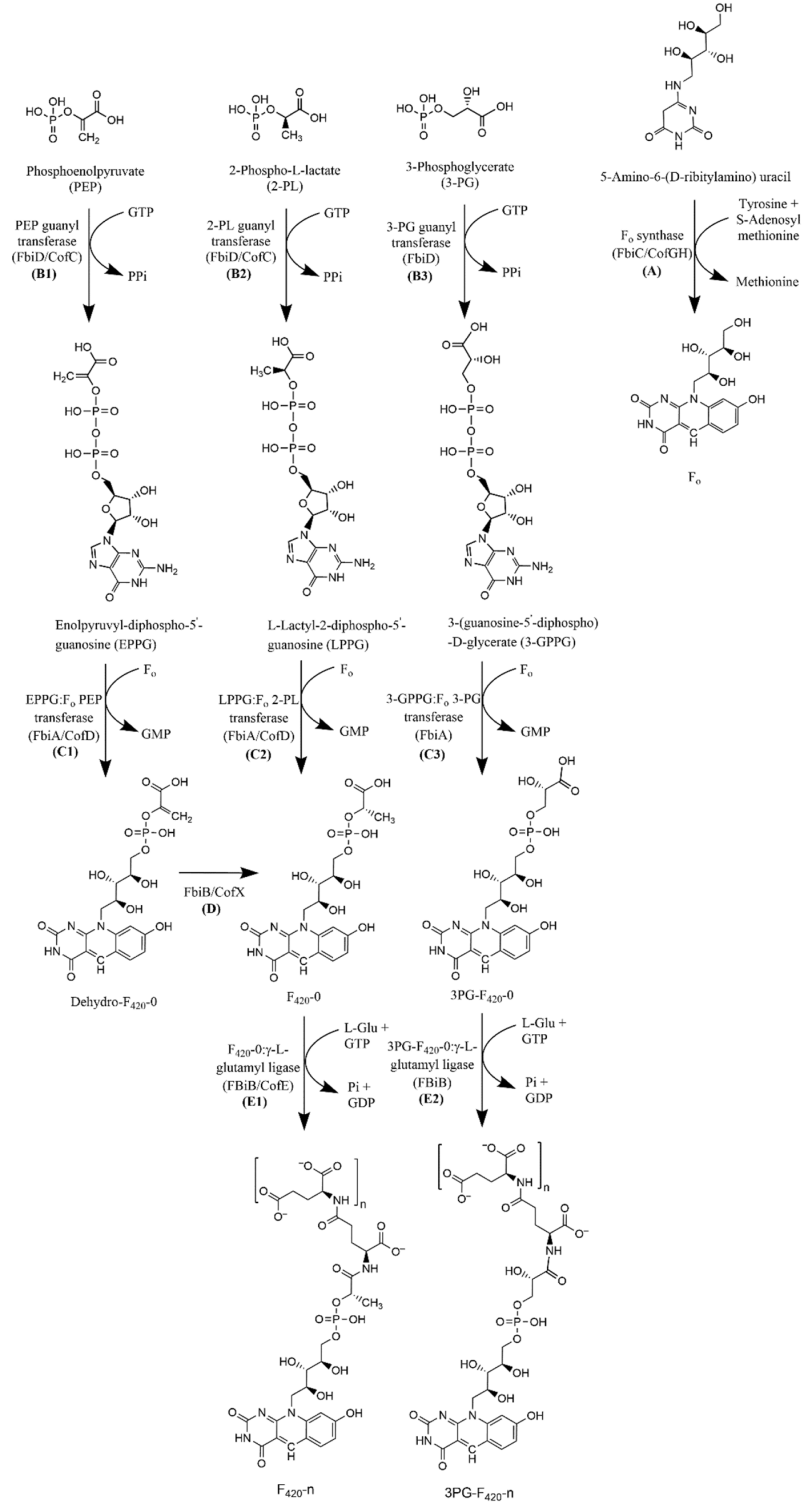
F_420_ biosynthesis pathway: F_420_ pathway has two branches, one branch begins with the formation of (**A**) F_o_ from 5-amino-6-(d-ribitylamino) uracil which is the intermediate of the riboflavin pathway, tyrosine and S-adenosyl methionine. The Reaction in this branch is catalysed by F_o_ synthase (FbiC/CofGH). Another branch of F_420_ is known to utilize different metabolites; PEP (FbiD/CofC)^18^ (**B1**); 2-PL (FbiD/CofC)^26^ (**B2**) and 3-PG(FbiD)^26^ (**B3**) producing EPPG, LPPG and 3-GPPG respectively. PEP and 3-PG are intermediates of the glycolytic pathway. In the next step LPPG (**C2**; FbiA/CofD) and 3-GPPG (**C3**; FbiA) together with F_o_ produce F_420_-0 and 3PG F_420_-0 respectively. In case of EPPG (**C1**) an intermediate Dehydro-F_420_-0^18^ is produced which is further converted to F_420_-0 (**D**). In the final step glutamylation of F_420_ is catalysed by CofE/FbiB (**E1** and **E2**) to produce either F_420_-n or 3PG-F_420_-n; number n depends on the F_420_ producing species. In this figure Cof genes are derived from archaea and Fbi genes are derived from bacteria. 3-PG derived F_420_ was observed recently in *P. rhizoxinica* bacteria^26^ and not yet been discovered in archaea.

For research applications, cofactor F_420_ is produced via fermentation of organisms that naturally produce it, in particular several species of *Mycobacteria*^15,18^. However, *Mycobacteria* are not well suited for large scale-production of the cofactor because they are not generally recognised as safe (GRAS) organisms, tend to form dense aggregated “clumps”, and are slow growing^19^. Two 13-step chemical syntheses of F_420_ isomers have been reported, differing only in the peptide linkage between the two glutamate residues. Both reported extensive use of protecting groups and low overall yield^20,21^. While improved syntheses of the deazaflavin moiety F_O_ have been reported (Fig. 1)^22^, it is unlikely that a full chemical-synthesis route to F_420_ will be economical due to low yield, poor atom economy and the instability of several intermediates. Although some F_420_-dependent enzymes have limited activity with F_O_, it is unlikely to be a suitable substitute due to poor kinetics^22^. More recently, an efficient chemoenzymatic approach to producing the F_420_ analogue F_O_P (phosphate group attached to F_O_) was reported and F_420_-dependent enzymes had substantially greater activity with F_O_P than F_O_, albeit still lower than with authentic F_420_^23^.

The bacterial F_420_ biosynthesis pathway proposed by Bashiri et al.^18^ was further updated in this study. F_O_ synthase (CofGH), one of the first steps in the F_420_ biosynthesis pathway, was found to be a radical SAM enzyme capable of generating two molecules of 5′-deoxyadenosine^24^. Therefore, F_O_ synthase accepts two molecules of *S*-adenosyl-l-methionine as substrate in addition to tyrosine, producing two molecules of l-methionine, one molecule of ammonia, and two molecules of 5'-deoxyadenosine (Fig. 1A).

Ideally, cofactor F_420_ would be produced via fermentation in a well-characterised microorganism, such as *Escherichia coli*, for which genetic and metabolic engineering tools are well developed and can be easily cultivated at large scale. Recently, we engineered the model laboratory bacterium *E. coli* to produce F_420_ via heterologous expression of biosynthetic genes sourced from *Mycobacterium smegmatis* (FbiD, FbiC, FbiB) and *Methanosarcina mazei* (CofD; equivalent to FbiA)^18^ (Fig. 1). The yield of cofactor F_420_ achieved in *E. coli* was 0.38 µmol/g DCW (grams of dry cell weight)^18^, which is comparable to yield obtained in wild type *Mycobacterium smegmatis*^25^. Thermodynamic analysis of cofactor F_420_ biosynthesis revealed that the overall pathway is energetically favourable, with the final steps being effectively irreversible. This suggests that yields attained could be improved upon through metabolic engineering^15^.

It has recently been shown that FbiD/CofC has species-specific substrate preferences (Fig. 1). FbiD from *Paraburkholderia rhizoxinica* prefers 3-phosphoglycerate (3PG)^27^ (Fig. 1, Step B3), *M. smegmatis and M. mazei* prefer phospho-enol pyruvate (PEP)^18,28^ (Fig. 1, Step B1), and *Methanococcus jannaschii* prefers 2-phospho-l-lactate (2PL)^29^ (Fig. 1, Step B2). It has been suggested that this step in the biosynthetic pathway may be particularly sensitive to the intramolecular concentration of its substrates, as thermodynamic analysis showed that this step is only just favourable in the forward direction^15^. The diversity of substrates used in various organisms may reflect the adaptation of this step to use highly abundant metabolites. Preliminary findings suggest that variation of phospho-carbohydrate moiety has less effect on F_420_-dependent enzymes than variations of tail length^30^,

Herein, we report that cofactor F_420_ biosynthesis in *E. coli* is heavily influenced by the carbon source. We used metabolic modelling to understand the underlying causes of carbon-source dependent differences in yield, which identified several potential bottlenecks. As a suitable genome-scale metabolic model was unavailable for production of F_420_ in *E. coli*, nor for any natural F_420_-producing organisms, we incorporated both the phospho-enol pyruvate-dependent and 3-phosphoglycerate-dependent F_420_ biosynthesis pathways into the iHK1487 genome-scale metabolic model for *E. coli* BL21^31^. The updated model (iEco-F420) was used to identify potential flux bottlenecks and to explore the theoretical limits of F_420_ production in this organism. Although the overall thermodynamics of the pathway are favourable, calculations revealed unfavourable energetics for the reaction catalysed by FbiD/CofC, which converts PEP and guanosine triphosphate into enolpyruvyl-diphospho-5′-guanosine^15^. Several strategies were explored to improve availability of PEP for this reaction. Through a combination of metabolic engineering and rational carbon source selection, we were able to improve the yield of cofactor F_420_ from 0.28 to 1.60 µmol/g DCW. The highest productivity observed with *E. coli* was a yield of 1.60 µmol/g DCW and culture time of 13 h (equivalent to 123 nmol/h/g DCW); this space–time yield is fourfold higher than is in recombinant *M. smegmatis,* for which the highest published yield of cofactor F_420_ achieved was 3.0 µmol/g DCW with culture time of 96 h (equivalent to 31 nmol/h/gDCW)^32^.

## Results and discussion

### The effect of different carbon sources on cofactor F_420_ yield and growth of *E. coli*

**Figure 2:**
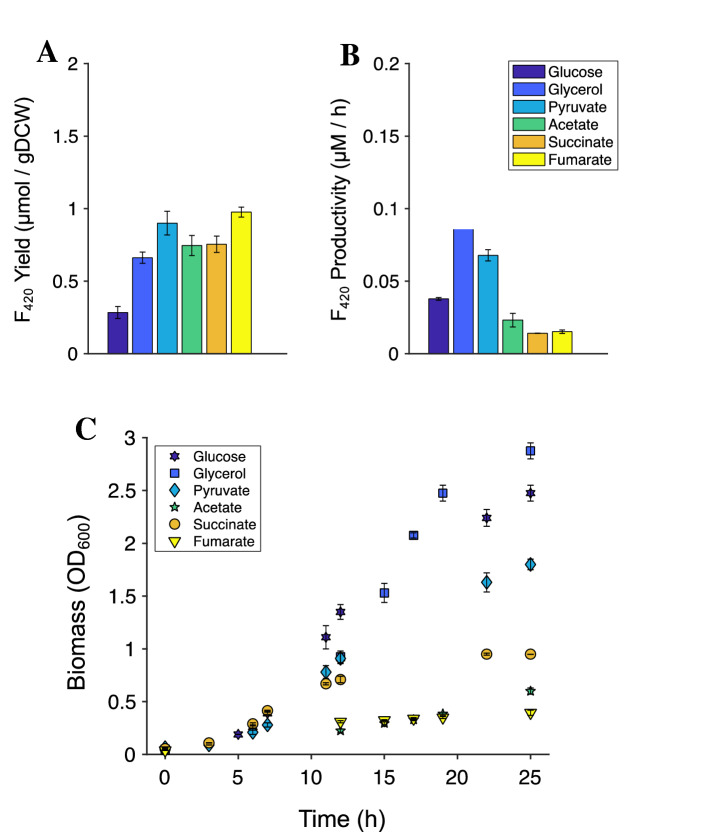
Cofactor F_420_ production and growth of *E. coli* expressing phospho-enol pyruvate-dependent F_420_ biosynthesis pathway using different carbon sources. (**A**) F_420_ yield (in µmol per grams of dry cell weight) at the end of exponential phase of *E. coli* growth. (**B**) Productivity of F_420_ (in µmol per liter per hour). (**C**) Growth of F_420_-producing *E. coli* (measured as optical density at 600 nm) over time. *E. coli* was cultivated in minimal media at 30 °C in shake flasks with different sole carbon sources. Error bars show standard errors of the mean of at least two replicates.

To investigate the effects of different carbon sources on the production of F_420_, we tested acetate, fumarate, glucose, glycerol, pyruvate, and succinate as carbon sources, as these carbon sources enter central metabolism at different points, have varied uptake mechanisms and therefore distinct bioenergetic consequences for the cell^33^. Pyruvate and fumarate, (followed closely by acetate and succinate) supported the greatest F_420_ production per gram of dry cell weight (DCW; Fig. 2A, B; Table 1). However, it should be noted that these carbon sources did not support high levels of biomass formation (Fig. 2C). Indeed, the cell density (measured as OD_600_) varied significantly by carbon source (Fig. 2C). With respect to overall productivity of F_420_ production (expressed as µmol F_420_/L/h), glycerol was the most productive carbon source (Fig. 2B); F_420_ yield with pyruvate was 0.90 µmol/g DCW which is close to the yield of the cofactor NADPH in *E. coli* of 1.3 µmol/g DCW^34^ (Fig. 2A). High F_420_ yield and productivity with pyruvate implicitly indicated the impact of this intracellular metabolite as well as its precursor, PEP, on F_420_ biosynthesis.

**Table 1:** Summary of the results obtained for engineered *E. coli* producing PEP-dependent or 3PG-dependent F_420_ using different carbon sources with and without overexpression of either PPS or PPCK.

Plasmid used	Carbon source	F_420_ yield (µmol/g DCW)	F_420_ concentration (µmol/L)	F_420_ productivity (µM/h)	Comments
pF420	Glucose	0.28 ± 0.01	0.39 ± 0.06	0.037 ± 0.001	PEP-dependent F_420_ biosynthesis pathway
pF420	Glycerol	0.66 ± 0.04	1.07 ± 0.06	0.096 ± 0.004	
pF420	Pyruvate	0.90 ± 0.08	0.91 ± 0.08	0.067 ± 0.004	
pF420	Acetate	0.74 ± 0.07	0.25 ± 0.04	0.023 ± 0.004	
pF420	Succinate	0.75 ± 0.06	0.40 ± 0.03	0.014 ± 0.000	
pF420	Fumarate	0.97 ± 0.03	0.22 ± 0.01	0.015 ± 0.001	
pF420 + PPS-pRSF	Glucose	0.26 ± 0.00	0.49 ± 0.00	0.050 ± 0.004	PEP-dependent F_420_ biosynthesis pathway along with over-expressed PPS (uninduced)
pF420 + PPS-pRSF	Glycerol	0.53 ± 0.10	0.76 ± 0.15	0.078 ± 0.001	
pF420 + PPS-pRSF	Pyruvate	1.61 ± 0.11	2.33 ± 0.16	0.175 ± 0.023	
pF420 + PPS-pRSF	Acetate	0.73 ± 0.01		0.021 ± 0.000	
pF420 + PPS-pRSF	Succinate	0.30 ± 0.02	0.19 ± 0.01	0.019 ± 0.002	
pF420 + PPS-pRSF	Fumarate	0.33 ± 0.00	0.08 ± 0.00	0.007 ± 0.000	
pF420 + PPS-pRSF	Glucose	0.54 ± 0.03	0.76 ± 0.05	0.076 ± 0.001	PEP-dependent F_420_ biosynthesis pathway along with over-expressed PPS (induced)
pF420 + PPS-pRSF	Glycerol	0.80 ± 0.03	0.94 ± 0.03	0.086 ± 0.001	
pF420 + PPS-pRSF	Pyruvate	0.97 ± 0.09	1.03 ± 0.10	0.070 ± 0.002	
pF420 + PPS-pRSF	Acetate	0.81 ± 0.06	0.43 ± 0.03	0.024 ± 0.002	
pF420 + PPS-pRSF	Succinate	0.32 ± 0.00	0.17 ± 0.00	0.016 ± 0.004	
pF420 + PPS-pRSF	Fumarate	0.27 ± 0.00	0.07 ± 0.00	0.005 ± 0.000	
pF420 + PPCK-pRSF	Glucose	0.19 ± 0.01	0.40 ± 0.01	0.049 ± 0.003	PEP-dependent F_420_ biosynthesis pathway along with over-expressed PPCK (uninduced)
pF420 + PPCK-pRSF	Glycerol	0.42 ± 0.04	1.09 ± 0.10	0.098 ± 0.039	
pF420 + PPCK-pRSF	Pyruvate	0.87 ± 0.04	1.56 ± 0.07	0.094 ± 0.008	
pF420 + PPCK-pRSF	Acetate	0.77 ± 0.05	1.47 ± 0.10	0.047 ± 0.005	
pF420 + PPCK-pRSF	Succinate	0.25 ± 0.02	0.27 ± 0.02	0.005 ± 0.000	
pF420 + PPCK-pRSF	Fumarate	0.22 ± 0.01	0.04 ± 0.00	0.002 ± 0.000	
pF420 + PPCK-pRSF	Glucose	0.27 ± 0.01	0.52 ± 0.02	0.035 ± 0.005	PEP-dependent F_420_ biosynthesis pathway along with over-expressed PPCK (induced)
pF420 + PPCK-pRSF	Glycerol	0.36 ± 0.06	0.70 ± 0.11	0.047 ± 0.005	
pF420 + PPCK-pRSF	Pyruvate	0.87 ± 0.07	1.19 ± 0.10	0.067 ± 0.002	
pF420 + PPCK-pRSF	Acetate	0.66 ± 0.04	0.95 ± 0.05	0.029 ± 0.005	
pF420 + PPCK-pRSF	Succinate	0.27 ± 0.04	0.27 ± 0.04	0.003 ± 0.000	
pF420 + PPCK-pRSF	Fumarate	0.25 ± 0.03	0.04 ± 0.01	0.002 ± 0.000	
pF420-3PG	Glucose	0.21 ± 0.01	0.53 ± 0.00	0.034 ± 0.003	3PG-dependent F_420_ biosynthesis pathway
pF420-3PG	Glycerol	0.38 ± 0.01	0.82 ± 0.02	0.048 ± 0.002	
pF420-3PG	Pyruvate	0.54 ± 0.01	0.88 ± 0.01	0.059 ± 0.000	
pF420-3PG	Acetate	0.32 ± 0.03	0.53 ± 0.02	0.014 ± 0.000	

### Phospho-enol pyruvate (PEP) is a key metabolite for F_420_ biosynthesis

**Figure 3:**
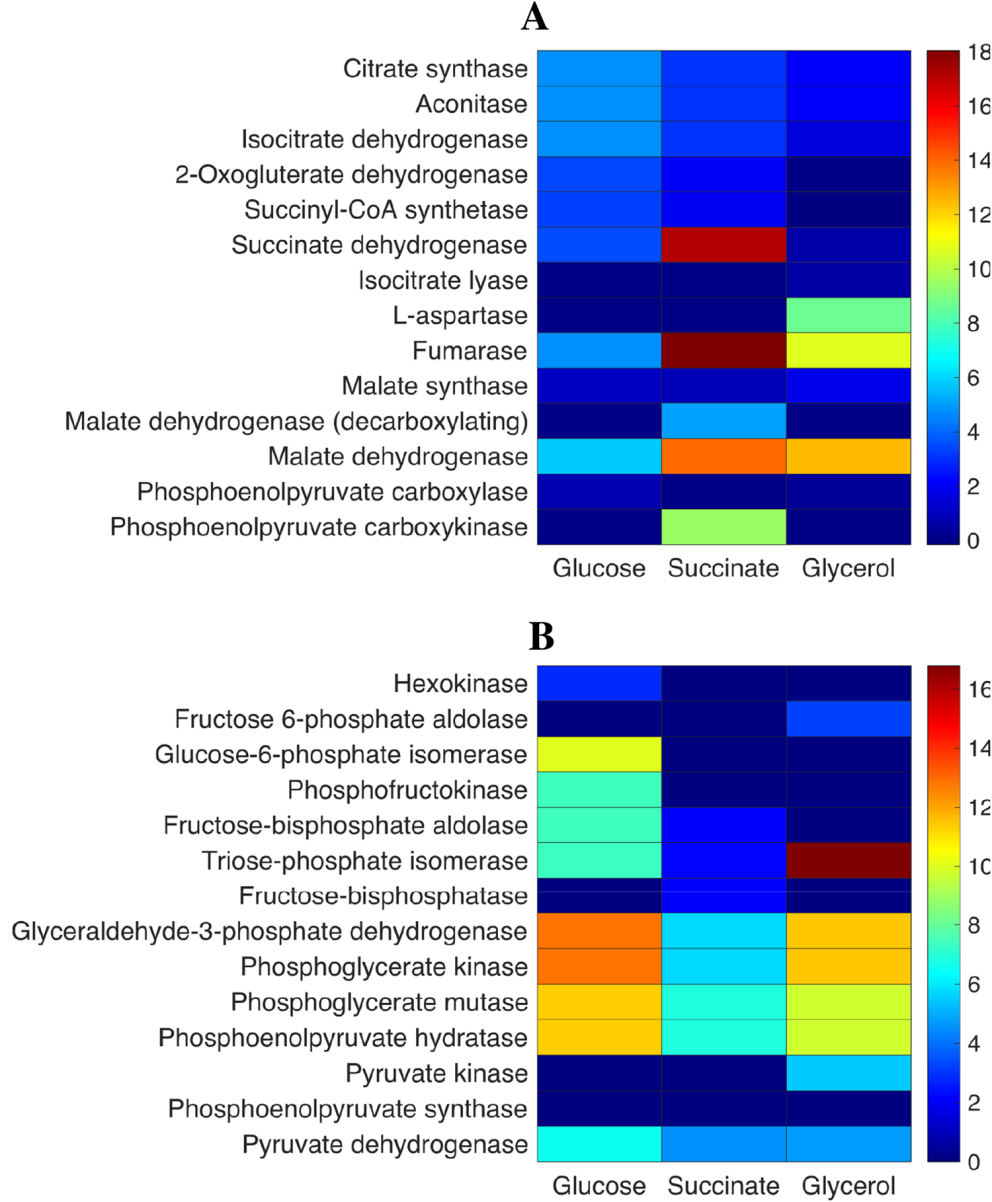
Flux balance analysis of TCA cycle and anaplerotic reactions of the TCA cycle (**A**) along with glycolysis/gluconeogenesis (**B**) pathways predicted by iEco-F420 metabolic model of *E. coli* for independent simulations using glucose, glycerol, or succinate as sole carbon sources (60 C-mol of carbon source). Objective is maximizing F_420_ production while maintaining growth at 30% of its max. Maintenance ATP requirements is fixed at 5.17 mmol/g DCW. Colormap shows absolute flux values in mmol/gDCW/hr. Fructose-bisphosphate aldolase, Triose-phosphate isomerase, Glyceraldehyde-3-phosphate dehydrogenase, and phosphoenolpyruvate hydratase are active in favor of gluconeogenesis pathway with succinate as the carbon source.

To systematically understand the effect of F_420_ biosynthesis on the distribution of flux through the entire metabolic network of the engineered *E. coli* grown with different carbon sources, we created and utilized the iEco-F420 metabolic model (see Methods) to compare flux profiles. Figure 3 summarises Flux Balance Analysis (FBA) results for two main pathways; glycolysis and the TCA cycle, for in silico growth with glucose, glycerol, and succinate as sole carbon sources, which were selected because of their different F_420_ productivity profiles. FBA predicted assimilation of 72% of glucose, as the sole carbon source, via the phosphoenolpyruvate (PEP): phosphotransferase system (PTS); all enzymes involved in glycolysis were active. Given the defined criteria, FBA predicted no flux through PEP synthase (PPS) or PEP carboxykinase (PPCK) indicating tight control over the pool of PEP during in silico growth with glucose (Fig. 3). These simulation results suggest a key role for PEP during F_420_ biosynthesis.

The metabolic model indicated that growth on succinate results in activation of the gluconeogenesis pathway and PPCK. With glycerol as the sole carbon source the upper glycolytic pathway was turned off (Fig. 3B), resulting in up to 27% higher overall ATP generation. On the other hand, fumarate was predominantly metabolised through aspartase since the glyoxylate shunt was highly active when glycerol was the carbon source, which leads to a reduction in total flux through TCA cycle. These modelling results explain the higher growth (Fig. 2C) and higher capacity for F_420_ production when engineered *E. coli* cells expressing the F_420_ biosynthetic pathway are grown with glycerol compared with glucose or succinate. Interestingly, with succinate as the carbon source, the iEco-F420 model predicted that pyruvate was produced mainly through malate dehydrogenase (decarboxylating) (Fig. 3A), leaving the PEP pool more accessible for incorporation into F_420_ production, consistent with the experimental yields. These results are consistent with the empirical growth experiments and also indicate a key role for PEP in controlling flux through the F_420_ biosynthesis pathway.

The iEco-F420 model contains 35 reactions consuming PEP: 19 are PEP-dependent phosphotransferases, 10 reactions participate in central carbon metabolism, two occur in cell envelope metabolism, two in tyrosine metabolism, and one in F_420_ biosynthesis (Supplementary File 1; Table S1). In an effort to increase the PEP pool, we used the model to test whether any of these competing reactions were dispensable in silico. However, single gene deletion in silico predictions suggested that removing the reactions involved in cell envelope and tyrosine metabolism would result in cell death.

**Figure 4:**
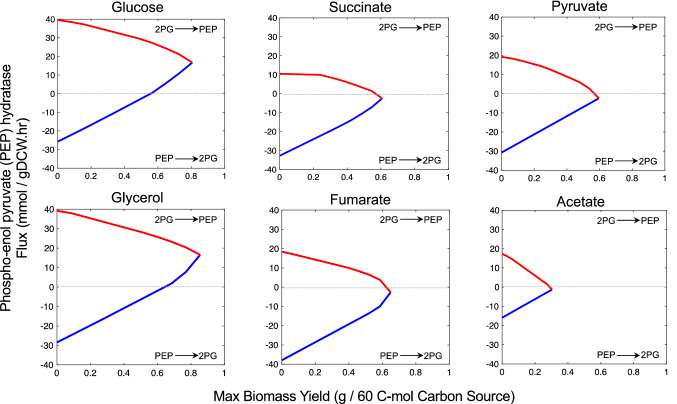
Flux variability analysis showing PEP utilization capacity represented by the flux through PEP hydratase as a function of cellular biomass yield for different carbon sources predicted by the iEco-F420 metabolic model of *E. coli*. Minimum and maximum fluxes are shown as blue and red, respectively. PEP: phosphor-enol pyruvate; 2PG: 2-phospho glycerate. Horizontal dashed lines do not correspond to any actual flux values; instead, they serve as hypothetical lines above which glycolysis drives carbon flow (2PG is metabolised to PEP).

We next performed flux variability analysis (FVA) for all reactions in the metabolic network, including the PEP-consuming reactions (Supplementary File 1; Tables S2–S13) to specifically explore flux variations in PEP-consuming/producing reactions as a result of maximization of flux through biosynthesis of F_420_. PEP hydratase (enolase) was chosen to interpret flux variations with respect to PEP availability for cellular growth *versus* F_420_ production. Figure 4 shows the flux profile of PEP hydratase using all six carbon sources. When glucose is the sole carbon source, PEP must be produced through glycolysis to meet cellular objective (i.e., maximizing growth). At maximum biomass (where the blue and red lines showing minimum and maximum fluxes meet in Fig. 4), PEP hydratase flux is positive, meaning that 2-phospho glycerate (2-PG) is fully metabolized to PEP. One engineering objective for increasing the heterologous production of F_420_ requires more carbon to be diverted into the target product rather than biomass, up to the point where the growth of the host is so negatively affected that it becomes uneconomical. When biomass yield drops to 80% of its maximum, for example, the minimum and maximum fluxes through PEP hydratase are still both positive, meaning that essential cellular processes take priority. As a result, 2-PG needs to be metabolized to provide stoichiometric requirements of PEP. However, at 50% of maximum biomass yield, the minimum flux (Fig. 4) through PEP hydratase becomes negative, meaning that the system is more relaxed to divert a portion of PEP for other processes including F_420_ production.

Unlike the flux predictions for PEP hydratase using glucose, PEP is significantly more available for processes other than cellular growth when the carbon source is succinate, fumarate or pyruvate, even at maximum biomass yields (Fig. 4). This is consistent with reports that glucose uptake in *E. coli* occurs primarily via the PTS, consuming up to 50% of the available PEP in cell^35,36^, thereby reducing its availability for F_420_ biosynthesis. Gluconeogenic carbon sources such as pyruvate, succinate, and fumarate increase intracellular PEP levels compared to glucose^33^ as their uptake is PEP-independent^36^. PEP hydratase flux variation with glycerol is the highest among other carbon sources, meaning that glycerol assimilation could potentially lead to greater flexibility in utilising PEP for F_420_ biosynthesis. However, glycerol uptake occurs through the glycolysis pathway and although its uptake requires half the energy (in form of ATP) of glucose, most of the PEP is still required for cellular activities rather than biosynthesis of F_420_. Nonetheless, glycerol remains a candidate carbon source for large-scale F_420_ production compared with glucose when maintaining high cell masses is essential because it allows for higher cellular mass yields while bypassing PTS-dependent PEP depletion. In the case of acetate, ATP-dependent acetate assimilation is the only route for producing acetyl-CoA, which is an essential precursor for the biosynthesis of most amino acids and fatty acids and therefore biomass yield drops significantly (Fig. 4). However, FVA for PEP hydratase indicates the feasibility of utilising PEP for non-cellular activities.

We measured intracellular PEP for engineered *E. coli* grown with glucose and glycerol to validate the model predictions. When glycerol was used as the sole carbon source, PEP and F_420_ levels were 1.43 and 1.82-fold higher, respectively, compared with when glucose was used as the carbon source. This difference was also borne out in the simulation data (Supplementary Table S1). These results, collectively, demonstrate that the choice of carbon source directly affects intracellular availability of PEP, which, in turn, influences F_420_ levels.

### Using 3PG as an alternative to PEP

As PEP is likely to be a flux-limiting metabolite, we explored the possibility of using an alternative metabolite in its place. Three different metabolites have been proposed to be incorporated in the sidechain of F_420_: PEP, 2-phospho-l-lactate and 3-phospho-d-glycerate^18,27,37^. While 2-phospho-l-lactate has not been observed in *E. coli*^18^, 3-phosphoglycerate (3PG) is a glycolytic pathway intermediate present in *E. coli* at 10 times the concentration of PEP^38^. Moreover, in the context of the iEco-F420 model, PEP-dependent F_420_ biosynthesis requires an additional FMN-dependent reduction step (the FbiB-dependent conversion of dehydro-F_420_-0 into F_420_-0) indicating that additional carbon would need to be diverted into FMN biosynthesis^28,39^. Preliminary evidence suggests that 3PG-F_420_, unlike F_O_ and F_O_P, is accepted as a cofactor by F_420_-dependent enzymes with similar kinetics to standard F_420_^30^. Given the relative abundance of 3PG, we investigated it as an alternative to PEP by substitution of *M. smegmatis* FbiD with that of *P. rhizoxinica.*

**Figure 5:**
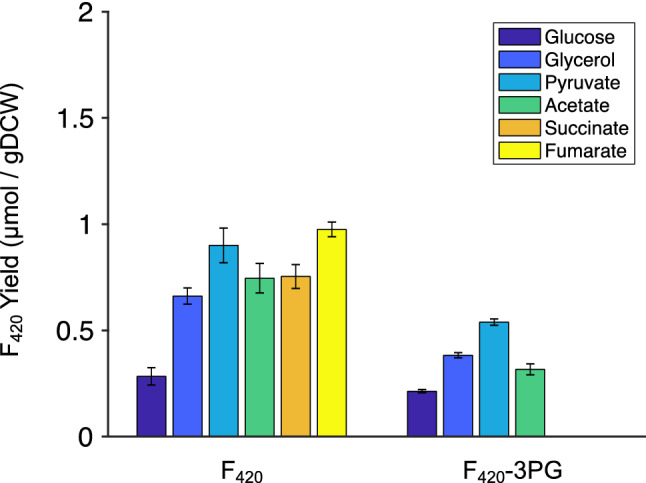
F_420_ yield (in µmol per grams of dry cell weight) at the end of exponential phase of *E. coli* growth with different carbon sources. 3PG-F_420_ and F_420_ refer to the biosynthesis of the cofactor F_420_ derived from 3-phospho glycerate and PEP, respectively, as the substrates of FbiD. Error bars show standard errors of the mean of at least two replicates.

Although 3PG is present at a higher intracellular concentration than PEP (1.5 mM *cf.* 0.18 mM)^38^ and is predicted to provide relatively similar maximum theoretical F_420_ yields (Supplementary Fig. S1), the experimentally determined yield of F_420_-3PG was found to be lower than for F_420_-PEP (Fig. 5). Moreover, no F_420_-3PG formation was observed with either succinate or fumarate as carbon source. This contrasts with the model predictions of feasible theoretical yields for F_420_-3PG with all carbon sources tested (Supplementary Fig. S1). It is possible that the *P. rhizoxinica* FbiD product, glyceryl-2-diphospho-5ʹ-guanosine, 3PG-F_420_-0 and/or its polyglutamated derivatives are poor substrates for the enzymes catalysing subsequent steps in F_420_ biosynthesis, which had been sourced from *Mycobacteria* and may have low specificity for 3PG containing F_420_ metabolites (Fig. 5).

### Over-expression of PEP synthase increases the yield of F_420_

Given that PEP is a limiting metabolite in F_420_ biosynthesis, we investigated whether production of PEP could be increased. Growth on fumarate and succinate is known to increase the expression of PEP-producing enzymes PPS and PPCK^40^ (Fig. 3A). Indeed, overexpression of PPS has been used to increase PEP concentrations in vivo^41,42^ to improve the yield of shikimic acid^43^, aromatic amino acids^42,44^ and lycopene^45^ biosynthesis. However, overexpressing PPS has been reported to negatively affect cell growth due to the excretion of pyruvate and acetate^42^.

**Figure 6:**
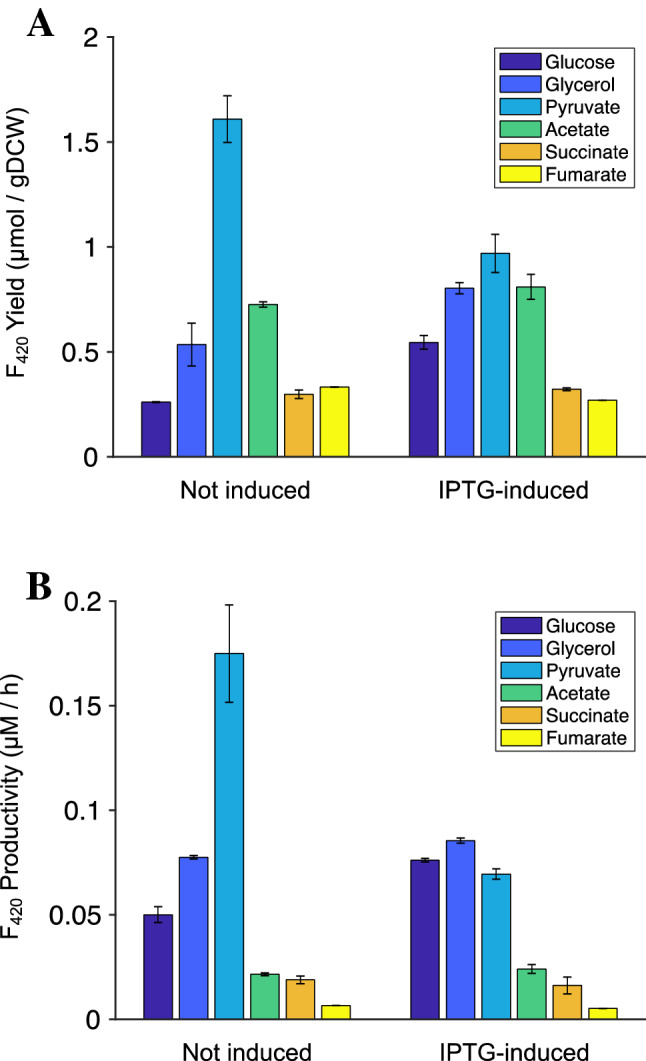
Effect of PPS over-expression on (**A**) F_420_ yield with and without IPTG (0.1 mM) induction; (**B**) F_420_ productivity (µM/h) with and without IPTG (0.1 mM). Leaky expression of PPS was observed without the addition of IPTG (Supplementary Fig. S3). *E. coli* was cultivated in a minimal media with different sole carbon sources as shown. Error bars show standard errors of the mean of at least two replicates.

In an attempt to increase intracellular PEP concentrations, we overexpressed PPS and PPCK from an IPTG-inducible expression plasmid and studied the effect on F_420_ yield. Consistent with previous reports^41^, overexpression of PPS resulted in growth inhibition. Therefore, to improve final biomass concentration, PPS was only induced once cell density (OD_600_) was greater than 1.0, which resulted in significant improvement in F_420_ yield. We tested overexpression of PPS when grown on different carbon sources, as shown in Fig. 6. Overexpression of PPS improved the yield of F_420_ from 0.27 to 0.54 µmol/g DCW using glucose and from 0.53 to 0.80 µmol/g DCW using glycerol*.* When grown on pyruvate, an F_420_ yield of 1.60 µmol/g DCW was observed without the addition of IPTG. With the addition of IPTG, the yield of F_420_ yield decreased to 0.90 µmol/g DCW. The yield of F_420_ also decreased after PPS induction when grown on succinate or fumarate (Fig. 6A). The pyruvate:PEP node of *E. coli* metabolism is highly regulated at both the transcriptional and metabolic levels^46^, it is possible that PPS is metabolically regulated during gluconeogenesis or that the reversable flux through PPS is being driven thermodynamically towards pyruvate formation when grown on gluconeogenic carbon sources. With glucose and glycerol, induction of PPS with IPTG resulted in significant improvement in the yield of F_420_ as compared to non-induced PPS. On the contrary, with pyruvate, non-induced PPS resulted in significantly higher yield and productivity of F_420_ compared to IPTG induced PPS. It may be that optimal PPS expression levels differ with different carbon sources. The highest yield of F_420_ obtained was 1.60 µmol/g DCW, with a productivity of 0.17 µmol/h, using pyruvate as carbon source with leaky expression of PPS.

**Figure 7:**
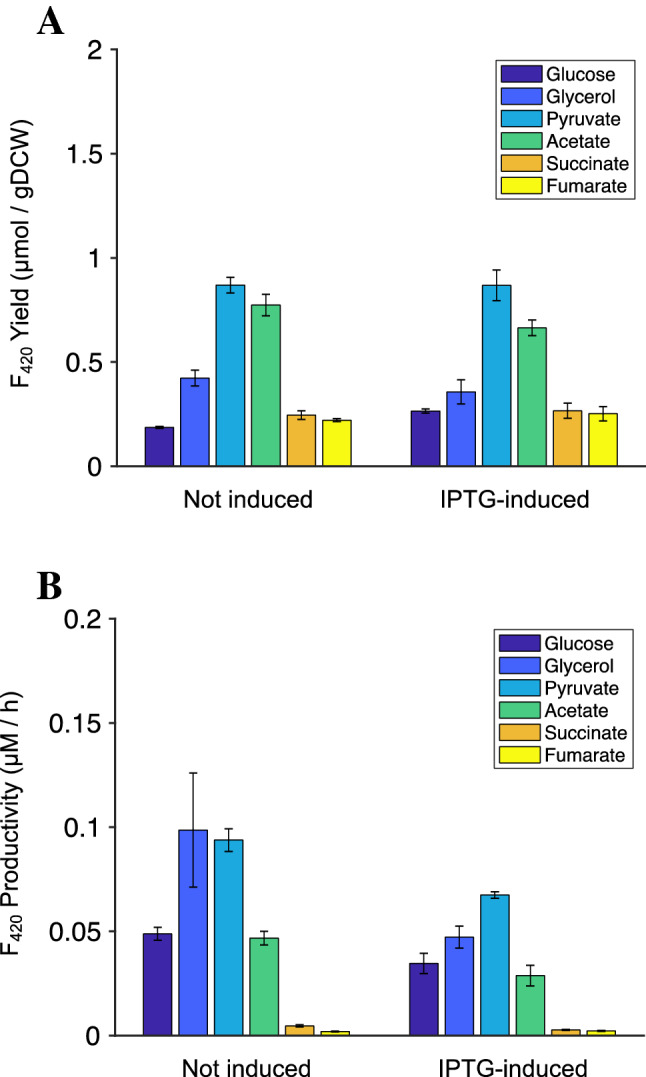
Effect of PPCK over-expression on (**A**) F_420_ yield with and without IPTG (0.1 mM) induction; (**B**) F_420_ productivity (µM/h) with and without IPTG (0.1 mM). Leaky expression of PPS was observed without the addition of IPTG (Supplementary Fig. S3). *E. coli* was cultivated in a minimal media with different sole carbon sources as shown. Error bars show standard errors of the mean of at least two replicates.

The impact of PPCK overexpression on F_420_ yield was also studied (Fig. 7). Unlike the expression of PPS, no improvement in F_420_ production was observed during PPCK overexpression. We confirmed the protein was expressed in soluble form (Supplementary Fig. S6). It is quite likely that we saw no difference in F_420_ concentration when PPCK was over-expressed because *E. coli* PPCK activity is metabolite controlled, either by the cellular PEP concentration or PEP:pyruvate ratio^46^. We therefore investigated the potential of uncontrolled PPCK overexpression using the iEco-F_420_ model. We explored the overall capability of the metabolic network to improve flux through FbiB (i.e., production of mature F_420_) by simulating over-expression of PPS or PPCK. The results, shown in Supplementary Fig. S2, indicate that by forcing a higher flux through PPS or PPCK, the maximum FbiB flux (shown by black arrows) drops unless it occurs at a non-zero flux through PPS or PPCK. These results indicate the maximum stoichiometric capacity for F_420_ biosynthesis as a result of over-expressing PPS or PPCK; however, the overall kinetics of the system and regulatory mechanisms for growth with different carbon sources would significantly influence F_420_ yields, in vivo. The experimental results (Fig. 6) confirmed improved F_420_ biosynthesis when using glucose and pyruvate as a result of PPS overexpression, in agreement with the simulation results shown in Supplementary Fig. S2 for these carbon sources. It should be noted that the simulation results of Supplementary Fig. S2 also demonstrate the potential impact of the type of transporter on the flux through CofE when over-expressing PPS or PPCK.

### The effect of time and carbon source on polyglutamate chain length

**Figure 8:**
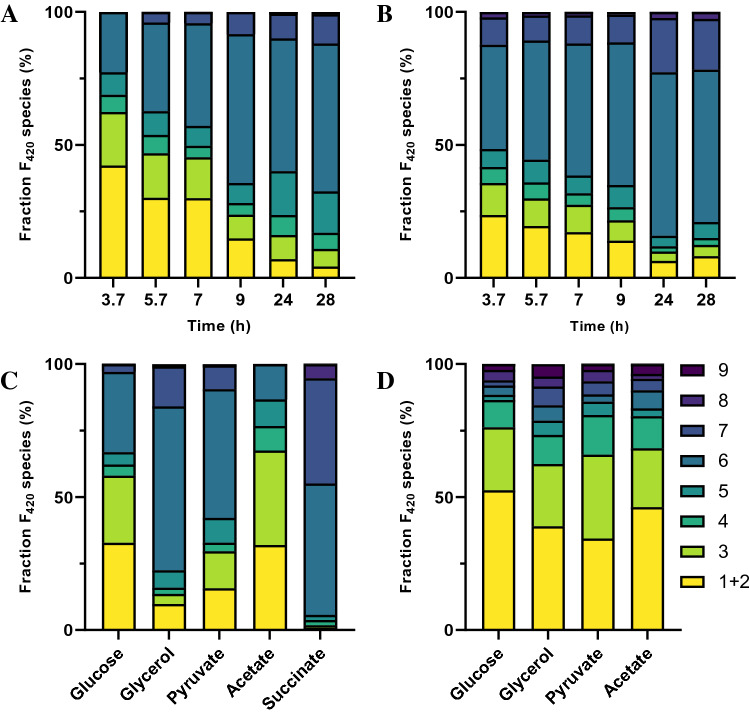
HPLC-FLD data for different glutamate residues attached to F_420_ at different time points after induction of F_420_ pathway using Glucose (**A**) and Glycerol (**B**). HPLC-FLD data for different glutamate residues attached to F_420_ (**C**) and 3PG -F_420_ (**D**) using different carbon sources at the end of exponential phase.

The final step in F_420_ biosynthesis is the addition of between one and nine glutamate residues to the F_420_-0 intermediate to yield F_420_-n (n: number of glutamate residues)^47,48^. What influences the tail length of F_420_ is still not clear, although in vitro analysis of F_420_-0:g-glutamate ligases from different organisms has revealed that they typically produce F_420_ species with polyglutamate chain lengths consistent with F_420_ obtained from the native organisms^48,49^. The number of glutamate residues influences the cofactor affinity of some F_420_-dependent enzymes; for example, the F_420_-dependent oxidoreductases MSMEG_2027, MSMEG_0777 and MSMEG_3380 from *M. smegmatis* reportedly having a high affinity for long chain F_420_ rather than shorter-chain F_420_^50^. Similar effects are seen with polyglutamylated folates and folate mimics^51–53^. Interestingly, F_420_-n composition changes with different growth phases of *Methanosarcina barkeri* and *M. mazei*^54^. We therefore investigated the composition of F_420_ during different growth phases of *E. coli.* The composition of F_420_-n at various time points is shown in Fig. 8. When grown with glucose or glycerol as the carbon source, *E. coli* initially produced short chain F_420_-(1–4) in higher proportions, which shifted over time to predominantly longer chain F_420_-(5–8) (Fig. 8A, B). CofE from *M. smegmatis* (the enzyme used in this system) has been shown to produce predominantly longer F_420_ species (5–8) in stationary phase^50^.

Interestingly, we found that the tail length distribution at the end of the exponential phase was influenced by the carbon source used (Fig. 8C, D). Growth on succinate yielded the highest proportion of long chain F_420_, with F_420_-(5–8) comprising > 90%. Glycerol has the next highest proportion of F_420_-(5–8) at > 80%, with glucose and acetate with the lowest levels of F_420_-(5–8) produces (< 30% and < 25%, respectively) (Fig. 8C). The iEco-F420 model was used to guide interpretation of carbon source-dependent tail length distribution. According to the cofactor biosynthesis pathway shown in Fig. 1, two molecules of GTP per molecule of glutamate are required to metabolise an F_420_ molecule with only one glutamate residue. Likewise, in an ideal case where all incoming carbon to the *E. coli* has to end up in F_420_ with only one glutamate residue, the iEco-F420 model predicted that the ratio of sum of fluxes through all glutamate-producing reactions (${v}_{glu}^{t}$) to sum of fluxes through all GTP-producing reactions (${v}_{gtp}^{t}$) has to be equal to two regardless of the type of carbon source. However, when the model was used to simulate F_420_ biosynthesis with chain length compositions observed experimentally, flux predictions suggested deviations in the ratio of ${v}_{glu}^{t}$ to ${v}_{gtp}^{t}$, which depends on the type of carbon source. Interestingly, the ratio of ${v}_{glu}^{t}$ to ${v}_{gtp}^{t}$ was predicted to be 1.731 and 1.772 using succinate and glycerol, respectively, showing the largest deviation for a ratio of two. On the other hand, the ratio of ${v}_{glu}^{t}$ to ${v}_{gtp}^{t}$ was predicted by the model to be 1.994, 1.960, and 1.873 using glucose, acetate, and pyruvate, respectively, explaining why the lowest proportion of long chain F_420_ was observed with these carbon sources.

3PG-F_420_ yielded significantly higher fraction of short chain F_420_-(1–4) > 70% (Fig. 8D) compared to PEP derived F_420_ irrespective of the carbon source used. This could be due to the difference in the kinetics of the enzymes for 3PG-F_420_ and PEP-F_420_.

**Figure 9:**
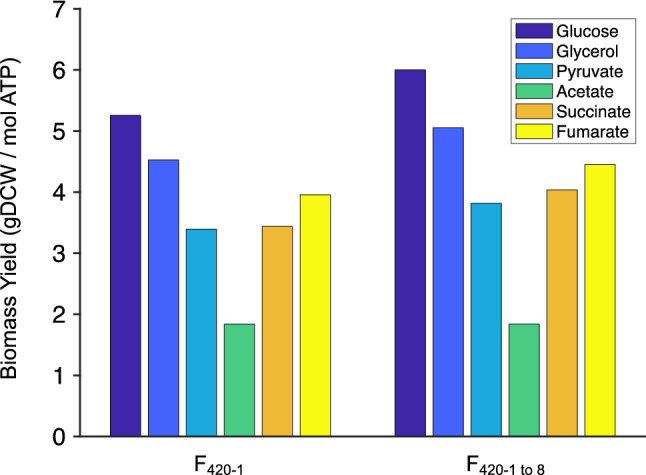
Theoretical biomass yields (at 30% maximum growth) with respect to total energy produced in the form of ATP predicted by the iEco-F420 metabolic model of *E. coli* BL21 simulated with different carbon sources. F_420_-1 indicates the yields for cells synthesizing cofactor F_420_ with only one glutamate tail, whereas F_420_-1 to 8 indicate those for cells synthesizing a mixture of F_420_ molecules with varying number of glutamate tails. The uptake of C-source was fixed to 60 C-mol in all simulations to account for differences in number of carbon atoms in C-sources.

The iEco-F420 metabolic model additionally provided some insights into the energetic differences in F_420_ biosynthesis with only one glutamate tail as well as with varying number of glutamate tails. For all carbon sources examined, yields were higher for F_420_ with only one glutamate than those for a mixture of F_420_ molecules with different chain-lengths. This is because at a fixed growth rate (i.e., constant cell mass yield), total energy production (in the form of ATP) is higher for biosynthesis of F_420_ with one glutamate than that for biosynthesis of a mixture of F_420_ molecules (Fig. 9). Based on the results illustrated in Fig. 9, glucose maintains the highest cell mass per mole of ATP produced by cells, which explains the low F_420_ yield from glucose compared to other carbon sources as shown in Fig. 2A. Assimilation of acetate as the sole carbon source requires the activation of ATP-dependent acetate kinase. Therefore, cells have to produce ATP in order to uptake carbon source for survival, which results in low growth rates (Fig. 2C) and maintaining the lowest cell mass yield per mole ATP produced among other carbon sources (Fig. 9) but, relatively high F_420_ yields (Fig. 2A; Supplementary Fig. S1). According to the modelling predictions, acetate might provide benefits from industrial perspective because, cells would be forced to produce ATP for fueling F_420_ production rather than for their growth.

## Conclusion

This work establishes that intracellular PEP concentration is the key limiting metabolic bottleneck for heterologous F_420_ biosynthesis in *E. coli*, at least when using biosynthetic enzymes, such as those from *M. smegmatis,* that natively use PEP. An updated metabolic model of *E. coli* incorporating the recombinant F_420_ biosynthetic pathway was developed and used to identify differences in metabolic flux distribution through the entire metabolic network including the central carbon metabolism using various carbon sources. This allowed us to identify, test and rationalize a number of approaches to improve F_420_ yield. Table 1 summarises the F_420_ yield, concentration, and productivity obtained using different strains and conditions in this study. In terms of productivity, glycerol was found to be the best carbon source for F_420_ production amongst those tested as it allowed the best balance between optimizing PEP concentration (compared to glucose) *vs.* slower growth (compared with acetate, succinate, etc.). We also examined whether alternative substrates for FbiD could be used to remove the reliance on PEP, showing that although 3PG is a viable substrate and is more abundant in *E. coli,* the use of the *P. rhizoxinica* FbiD did not result in higher 3PG-F_420_ yields (compared with F_420_), presumably because of the downstream enzymes, which are sourced from *M. smegmatis*, have a preference for F_420_-metabolites over 3PG-F_420_ metabolites. Replacing other enzymes in the pathway with those from *P. rhizoxinica* may be a worthwhile strategy to improve 3PG-F_420_ yield, as the theoretical yield of 3PG-F_420_ is similar to that of PEP-derived F_420_ and strategies have already been developed to improve 3PG availability by increasing glycolytic flux by knocking out the *zwf* gene involved in first step of pentose phosphate pathway^55^. Other strategies that we tested to increase F_420_ yield included increasing PEP production through over expression of PPS; this produced the highest productivity with pyruvate. Our results also indicate that F_420_ composition and concentration in *E. coli* is comparable to some of the best natural producers such as *M. smegmatis.* The F_420_ yield obtained with *M. smegmatis* is approximately 0.30 µmol/g DCW in wild type^25^ and 3.0 µmol/g DCW in engineered *M. smegmatis*^32^. However, cultivation of *M. smegmatis* in shake flasks takes up to 96 hours^32^, while engineered *E. coli* cultivation takes 16 h (this study). This results in space–time yield of 114 nmol/h/gDCW for the recombinant *E. coli* system, *vs.* 31 nmol/h/gDCW for the recombinant *M. smegmatis* system^32^. This increased yield is in addition to the many other advantages of using *E. coli,* including less expensive antibiotics (ampicillin *vs.* hydromycin), reduced safety risks and thus greater accessibility to the technology, and the fact that *E. coli* is a much more widely used strain for protein engineering and DNA modification. By systematically optimizing growth and production conditions in *E. coli*, we have created a system that should make production of F_420_ more economical at an industrial scale and the study of F_420_-depenent enzymes more accessible, improving on the previous attempts for F_420_ production through the use of more exotic and challenging bacterial species, such as *M. smegmatis.*

## Materials and methods

### Bacterial strain, vector and media composition

*E. coli* BL21 DE3 (New England Biolabs) was used for protein expression and DH5α (New England Biolabs) strain was used for plasmid propagation. LB media consisting of 10 g/L tryptone, 5 g/L yeast extract and 10 g/L of NaCl was used for the plasmid propagation and cloning, 15 g/L of agar was added to prepare LB agar plates. *E. coli* protein expression and F_420_ production studies were done in M9 minimal media consisting of 6.78 g/L Na_2_HPO_4_, 3 g/L KH_2_PO_4_, 1 g/L NH_4_Cl, 2.5 g/L NaCl, 100 × trace elements solution (1.667 g/L FeCl_3_∙6H_2_O, 0.018 g/L ZnSO_4_∙7H_2_O, 0.012 g/L CuCl_2_∙2H_2_O, 0.012 g/L MnSO_4_∙H_2_O, 0.018 g/L CoCl_2_∙6H_2_O, and 2.225 g/L Na_2_EDTA∙2H_2_O), 0.246 g/L MgSO_4_∙7H_2_O, 0.011 g/L CaCl_2_, and 5 g/L of glucose or 0.167 C-mol of different carbon sources with the final pH of 7.3. Chloramphenicol (25 µg/ml), kanamycin (50 µg/ml) and ampicillin (100 µg/ml) were added where appropriate.

*E. coli* was cultivated in shake flasks at 30 °C with shaking at 200 rpm. The F_420_ biosynthesis genes in pSB1C3 plasmid were induced with 200 ng/ml of tetracycline. *E. coli* with pRSF duet plasmid was induced with IPTG (0.1 mM) for the expression of PPS and PEP carboxykinase (PPCK) enzymes. The biomass was measured using optical density at 600 nm.

### Plasmid construction

The synthesis of the plasmid expressing the F_420_ biosynthesis pathway operon was described in Bashiri et al*.*^18^. The pathway consists of CofD (Accession number: Q8PVT6), CofE (A0QTG1), CofC (A0QUZ4) and CofGH (NC_008596) genes under the control of the tetracycline-inducible promoter BBa_R0040^56^ and the artificial terminator BBa_B1006^57^. The F_420_ biosynthesis operon had been previously synthesized by GenScript and cloned in to pSB1C3 containing the constitutive tetracycline repressor cassette BBa_K145201 with *Eco*RI/*Xba*I and *Pst*I/*Spe*I restriction enzymes, plasmid construction is explained in Bashiri et al*.*^18^. This construct, hereafter referred to as pF420, enables production of F_420_ to be induced by the addition of tetracycline. All the gene sequences except CofD were obtained from *Mycobacterium smegmatis,* CofD gene sequence was obtained from *Methanosarcina mazei.* All the genes were codon optimized for expression in *E. coli* BL21 DE3 strain. All F_420_ proteins were FLAG tagged and soluble expression of F_420_ pathway proteins was confirmed using western blot^18^. In order to produce 3PG-F_420_, the *M. smegmatis* CofC homologue was replaced by the homologue from *Paraburkholderia rhizoxinica* (E5ASS2)*.* This F_420_ biosynthesis operon was synthesised (Biomatik) in two fragments (P_rhizo_3PG-F420-1 and P_rhizo_3PG-F420-2), P_rhizo_3PG_F420-1 was a 3.2 kb flanked with *Eco*RI and *Bam*HI restriction sites in pUC57, P_rhizo_3PG-F420-2 was a 3.6 kb flanked with *Bam*HI and *Pst*I restrictions sites in vector pUC57. Three-way ligation was performed to ligate P_rhizo_3PG-F4201 (cut with *Eco*RI and *Bam*HI), P_rhizo_3PG-F4202 (*Bam*HI and *Pst*I) and pF420 (cut with *Eco*RI and PstI). The ligation resulted in plasmid pF420-3PG (Supplementary Table S2).

The *ppsA* gene encoding PPS from *E. coli* (P23538) and *pck* gene encoding PPCK from *E. coli* (B5YTV3) were synthesized and cloned into pETCC2^58^ plasmid flanked by *Nde*I and *Bam*HI restriction sites (Twist Bioscience) to produce PPS-pETCC2 and PPCK-pETCC2 plasmids (Supplementary Table S2). pF420 have mutated pUC57 origin of replication which causes high copy number^59^ and pETCC2 have pBR322 origin of replication , which are incompatible for co-transformation. Therefore, genes encoding PPS and PPCK were cloned into pRSF duet in order to have replicative compatibility with pF420 vector. pETCC2 and pRSF duet plasmids were digested with restriction enzymes *Nde*I and *Bam*HI, the genes were gel purified from pETCC2 digestion and were ligated to pRSF duet plasmid. The pRSF duet plasmid containing the genes encoding PPS (PPS-pRSF) or PPCK (PPCK-pRSF) were used to transform BL21 DE3 containing pF420 plasmid using electroporation. Transformed cells were selected on LB agar plates supplemented with chloramphenicol and kanamycin. Supplementary Table S2 summarises the list of plasmids used in this study.

Expression of F_420_ pathway proteins in pF420 had been confirmed previously using immunoblotting. The procedure is explained in detail by Bashiri et al*.*^18^. Soluble expression of PPS, PPCK and F_420_-3PG operon proteins was confirmed through SDS-PAGE gel (Supplementary Fig. S3).

## Analytical methods

### F_420_ detection, quantification and chain length measurement

Production of F_420_ in *E. coli* and its detection was confirmed using LC–MS, as previously reported^18^. For the quantification of F_420_ from *E. coli,* 1 ml of sample from shake flask cultivation was taken and centrifuged at 10,000*g* for 2 min. The pellet was resuspended in 120 µl of 75% ethanol, boiled for 3 min at 94 °C to lyse the cells, resuspension was centrifuged at 10,000*g* for 2 min and the fluorescence of 100 µl supernatant was measured in a SpectraMax M3 (Molecular Devices) 96-well plate spectrofluorometer (excitation at 420 nm and emission at 480 nm). Fluorescence correlated directly with F_420_ concentration in the cell lysate. F_420_ amount relative to the biomass concentration was obtained in fluorescence (Units)/Biomass (OD_600_). The correlation between Biomass (OD_600_) and dry cell weight (DCW) was 0.56 g/L DCW for OD_600_ of 1.0. There was a linear correlation between absorbance at 420 nm and fluorescence (420 nm excitation and 480 nm emission). The fluorescence values of the cell lysate were converted to absorbance, and the extinction coefficient of 41.4 mM^−1^/cm was used^60^ to convert fluorescence unit of cell lysate to mM of F_420_ in cell lysate. Using these parameters fluorescence (units)/Biomass (OD) was converted to µmol F_420_/g DCW.

Analytical separation of F_420_ species based on the length of its glutamate tail is shown in S Supplementary Fig. S4 and was achieved with an ion-paired reverse phase HPLC-FLD protocol as reported previously^61^. The supernatant was run on an Agilent 1200 series HPLC system equipped with an Agilent fluorescence detector and an Agilent Poroshell 120 EC-C18 2.1 × 50 mm 2.7 mm column. The system was run at a flow rate of 0.5 ml/min and the samples were excited at 420 nm and emission was detected at 480 nm. A linear gradient of two buffers were used: Buffer A, containing 20 mM ammonium phosphate, 10 mM tetrabutylammonium phosphate, pH 7.0. Buffer B, 100% acetonitrile. A gradient was run from 25 to 40% buffer B as follows: 0–1 min 25%, 1–10 min 25%–35%, 10–13 min 35%, 13–16 min 35–40%, 16–19 min 40%–25%.

Intracellular PEP was measured using single ion monitoring method (SIM) in single quad (Agilent 6120). Two buffers were used, Buffer A containing 10 mM ammonium formate with pH adjusted to 4.0 and Buffer B containing 100% acetonitrile, isocratic flow with 25% buffer B was used with flow rate of 0.5 ml/min using Agilent Poroshell 120 EC-C18 2.1 × 50 mm 2.7 mm column. 10 µl of sample was injected and SIM method was used to detect mass of 167 *m/z* in negative mode in order to estimate PEP concentration in cell lysate.

### Metabolic network model of *E. coli* BL21

The iHK1487 metabolic model of *E. coli*^31^, containing 1487 genes, 2701 reactions, and 1164 metabolites, was used as the scaffold to integrate F_420_ biosynthesis pathway in this study. 360 reactions were found to be mass or charge imbalanced in the model, of which 338 reactions were demand or exchange reactions and one was biomass reaction, and therefore, were excluded from further corrections. Of the remaining 21 imbalanced reactions, 12 were involved in the lipopolysaccharide and cell envelope biosynthesis/recycling pathways, four were in alternate carbon metabolism pathway, three were in capsular polysaccharide biosynthesis/recycling pathway, and two were transporters (Supplementary File 1; Table S14). 14 imbalanced reactions were corrected by balancing protons, correcting chemical formula, or modifying participating metabolite(s) (see Supplementary File 1 for details). For example, four new metabolites were amended to the metabolic model to represent balanced core oligosaccharide lipid A molecules. The remaining seven imbalanced reactions could not be further resolved due to the lack of metabolite and/or enzyme specificity, rendered non-essential after flux analysis.

The bacterial F_420_ biosynthesis pathway proposed by Bashiri et al.^18^ was added to the original metabolic model to enable prediction of F_420_ production by engineered *E. coli*. This updated model was named iEco-F420. F_O_ synthase in the iEco-F420 model was modified by adding two molecules of *S*-adenosyl-l-methionine to the reactant side, and adding two molecules of l-methionine, one molecule of ammonia, and two molecules of 5′-deoxyadenosine to the product side of the catalysing reaction. Moreover, the updated model allows for analysing the biosynthesis of cofactor F_420_ with up to eight glutamate residues (through l-glutamate:coenzyme F_420_ ligase, CofE). For that, either an F_420_ molecule with one glutamate residue or a stoichiometric combination of F_420_ molecules with varying glutamate tails can be set as the target to analyse their flux and biosynthesis profile. Furthermore, the iEco-F420 model contains F_420_-dependent formate dehydrogenase^62^, F_420_-dependent G6P dehydrogenase^63^, F_420_-NADP oxidoreductase^64^, F_420_-dependent oxidoreductase^50^, and F_420_-reducing hydrogenase^65^ allowing for the analysis of cofactor F_420_ recycling and regeneration within the metabolic network of engineered F_420_-producing *E. coli*.

Integrating the F_420_ biosynthesis pathway along with correcting imbalances resulted in the iEco-F420 metabolic model of *E. coli* with 26 new metabolites and 43 new reactions. The updated metabolic model is available in Excel format in Tables S15 and S16 of Supplementary File 1. All reaction fluxes are in mmol/gDCW-h except for the reaction representing cell biomass formation that is expressed in h^−1^. The M9 minimal medium composition was used to constrain the input of nutrients in the updated model. Independent simulations were run using glucose, glycerol, pyruvate, acetate, fumarate, or succinate as sole carbon sources (60 C-mol of carbon source). The uptake of C-source was fixed to 60 C-mol in all simulations to account for differences in number of carbon atoms in C-sources. The objective function was to maximize F_420_ production, while maintaining growth at 30% of its maximum to represent in vivo growth conditions. Maintenance ATP requirements were fixed at 5.17 mmol/g DCW and the minimum oxygen uptake was set to 18.5 mmol/g DCW/h. For simulating in vivo over-expression of PPS and PPCK experiments, flux through PPS or PPCK was fixed in each run by constraining their lower and upper bounds to a value between zero and 70 mmol/g DCW/h, whereas zero represents no over-expression. Only one carbohydrate transporter was allowed to be active in each of these simulation runs. The model was assembled in a format compatible for flux balance analysis^66^. FBA optimization problems were solved by GNU Linear Programming Kit (GLPK) (http://www.gnu.org/software/glpk/) solver in MATLAB using COBRA toolbox^67^. Flux variability analysis (FVA) was performed to obtain range of fluxes under optimal growth conditions as described previously^68^.

## Supplementary Information

Supplementary Information 1.

Supplementary Information 2.

## Data Availability

The datasets generated during and/or analysed during the current study are available either as supplementary files or from the corresponding author on reasonable request.
