# Supplementary material for: Improved production of the non-native cofactor F420 in Escherichia coli
Source: Sci Rep. 2021 Nov 5;11:21774. doi: 10.1038/s41598-021-01224-3 (PMC8571402; doi:10.1038/s41598-021-01224-3)
Supplement: Supplementary file 1 — Supplementary Information 1. [file 41598_2021_1224_MOESM1_ESM.docx]

**Improved production of the non-native cofactor F_420_ in *Escherichia coli* – Supplementary materials**

Mihir V. Shah^1,2^, Hadi Nazem-Bokaee^1,2^, James Antoney^2,3^, Suk Woo Kang^2,3^, Colin J. Jackson^2,3^, Colin Scott^1,2^.

*^1^Biocatalysis and Synthetic Biology Team, CSIRO Land & Water, Black Mountain Science & Innovation Precinct, Canberra, Australia*

*^2^Synthetic Biology Future Science Platform, Black Mountain Science & Innovation Precinct, Canberra, Australia*

*^3^Research School of Chemistry, Australian National University, Canberra, Australia*

**Supplementary Figure 1.** Maximum theoretical yields of cofactor PEP-F_420_-1 , PEP-F_420_-1 _to 8_, or 3PG-F_420_ biosynthesis at 30% of maximum growth predicted by the iEco-F420 metabolic model of *E. coli* BL21 simulated with different carbon sources.

**Supplementary Figure 2**. Flux through CofE as a function of increasing flux through PPS or PPCK predicted by iEco-F420 model simulated with different carbon sources. A black arrow in each plot shows maximum theoretical flux through CofE. If multiple transporters for a carbon source exist, independent simulations are performed and results are shown in different colours


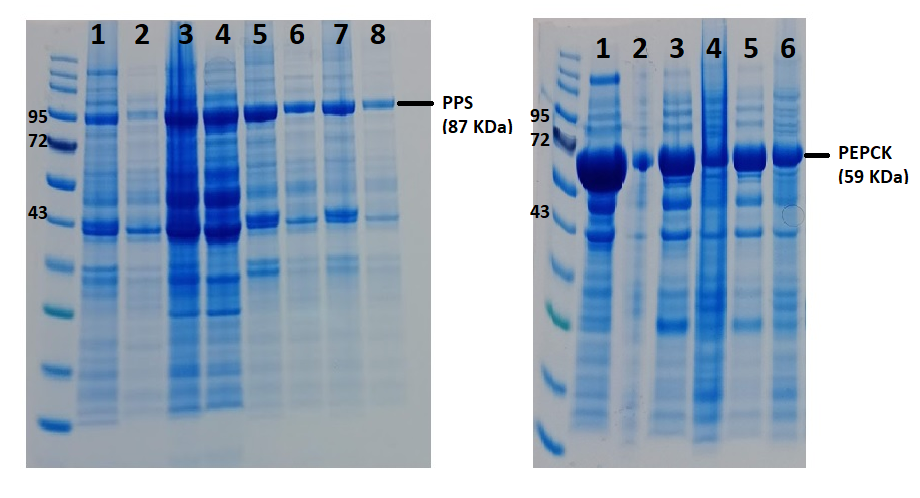


1. (B)

**Supplementary Figure 3**: SDS-PAGE analysis of the expression of PPS (A) and PEPCK (B). IPTG was added at a final concentration of 0.1 mM.

(A) 1. Insoluble protein with glucose as carbon source without adding IPTG; 2. Soluble protein with glucose as carbon source without adding IPTG; 3. Insoluble protein using glycerol as carbon source without adding IPTG; 4. Soluble protein using glycerol as carbon source without adding IPTG; 5. Insoluble protein using glycerol as carbon source after IPTG induction; 6. Soluble protein using glycerol as carbon source after IPTG induction; 7. Insoluble protein using glucose as carbon source after IPTG induction; 8. Soluble protein using glycerol as carbon source after IPTG induction.

(B) 1. Soluble protein using glucose as carbon source after IPTG induction; 2. Insoluble protein using glucose as carbon source after IPTG induction; 3. Soluble protein using glucose as carbon source after IPTG induction; 4. Insoluble protein using glucose as carbon source after IPTG induction; 5. Soluble protein using glucose as carbon source after IPTG induction; 6. Insoluble protein using glucose as carbon source after IPTG induction.

**Supplementary Figure 4.** Preparative separation of F_420_ species by size-exclusion chromatography. Samples were dissolved in Milli-Q water loaded on a HiLoad 16/60 Superdex 30 pg (GE Healtcare) equilibrated with 0.3 M ammonium bicarbonate pH 8.5. Composition of each 2 mL fraction was determined by mass spectrometry. The dashed line separates peak areas plotted against the left and right Y axes.

**Supplementary Table 1**. Flux values (mmol/gDCW/hr) through PEP and F_420_ accumulation using glucose vs. glycerol predicted by the iEco-F420 metabolic model. The ratio of F_420_ and PEP fluxes (in fold change) predicted using glycerol to that using glucose were also given. To allow simulating intracellular PEP measurements, it was assumed that PEP could be accumulated in a contained space inside cytosol by adding a reaction driving PEP to that hypothetical space (i.e., PEP[within the entire cytoplasmic space] -> PEP[within a small contained space in cytosol]). FBA was performed with the objective of finding maximum flux through PEP in that space.

|  | Glucose | Glycerol | Fold change (Glycerol/Glucose) |
| --- | --- | --- | --- |
| F_420_ | 1.15046892 | 1.4132161 | 1.22838268 |
| PEP | 16.7607219 | 19.9826416 | 1.19223036 |

**Supplementary Table 2. Plasmids used in this study**

| **Name** | **Description** | **Source** |
| --- | --- | --- |
| pF420 | pUC ori, pSB1C3 plasmid backbone, Cm^R^, PEP utilising F_420_ operon, Tet inducible | *^12^* |
| PPS-pETCC2 | pBR322 ori, PPS gene synthesised and cloned into pETCC2 (Twist Bioscience), Amp^R^, IPTG inducible | This study, pETCC2*^51^* |
| PPCK-pETCC2 | pBR322 ori, PPCK gene synthesised and cloned into pETCC2 (Twist Bioscience), Amp^R^, IPTG inducible | This study |
| PPS-pRSF | RSF1030 ori, PPS gene insert cloned from PPS-pETCC2 into pRSF-duet plasmid, Kan^R^, IPTG inducible | This study |
| PPCK-pRSF | RSF1030 ori, Kan^R^, PPCK gene insert cloned from PPCK-pETCC2 into pRSF-duet plasmid, Kan^R^, IPTG inducible | This study |
| P_rhizo_3PG-F420-1 | pUC ori, Part of F420-3PG operon synthesised and cloned into pUC57-Kan (Biomatik), Kan^R^ | This study |
| P_rhizo_3PG-F420-2 | pUC ori, Part of F420-3PG operon synthesised and cloned into pUC57-Kan (Biomatik), Kan^R^ | This study |
| pF420-3PG | pUC ori, Obtained from ligating F420-3PG-1, F420-3PG-2 and pSB1C3 plasmid backbone, Cm^R^, 3PG utilising F_420_ operon, Tet inducible | This study |
